# Risk and prediction of job burnout in responding nurses to public health emergencies

**DOI:** 10.1186/s12912-024-01714-5

**Published:** 2024-01-17

**Authors:** Lu Wang, Xiaohong Zhang, Meng Zhang, Lei Wang, Xiaoru Tong, Na Song, Junyi Hou, Juan Xiao, Hong Xiao, Tingting Hu

**Affiliations:** Nursing Department, Xiangyang Central Hospital, Affiliated Hospital of Hubei University of Arts and Science, Xiangyang, Hubei China

**Keywords:** Nurses, PTSD, Burnout, Risk factors, Nomogram

## Abstract

**Background:**

In public health emergencies, nurses are vulnerable to adverse reactions, especially job burnout. It is critical to identify nurses at risk of burnout early and implement interventions as early as possible.

**Methods:**

A cross-sectional survey of the hospitals in Xiangyang City was conducted in January, 2023 using stratified cluster sampling. Anonymized data were collected from 1584 working nurses. The Impact of Events Scale-Revised (IES-R) and the Chinese version of the Maslach Burnout Inventory-General Survey (MBI-GS) were used to evaluate the post-traumatic stress disorder (PTSD) and burnout of nurses in public health emergencies. Logistic regression analysis was established to screen for risk factors of burnout, and a nomogram was developed to predict the risk of burnout. A calibration curve and the area under the receiver operating characteristic (ROC) curve were used to validate the nomogram internally.

**Results:**

This study showed that only 3.7% of nurses were completely free of PTSD during a public health emergency. We found that PTSD varied by age, marital status, procreation status, length of service, employee status, and whether working in the ICU. The nurses aged 30 ~ 40 years old, single, married without children, non-regular employees, worked for less than three years or worked in the ICU had higher levels of PTSD. Regarding the prevalence of burnout, 27.4%, 48.5%, and 18.6% of nurses had a high level of emotional exhaustion (EE), depersonalization (DP), and diminished personal accomplishment (PA), respectively. There, 31.1% of nurses had more than two types of job burnout. The number of night shifts, the type of hospital, marital status, and the severity of PTSD were all associated with higher rates of exhaustion among nurses. As a graphical representation of the model, a nomogram was created and demonstrated excellent calibration and discrimination in both sets (AUC = 0.787).

**Conclusions:**

This study confirmed the PTSD and burnout are common problems for in-service nurses during public health emergencies and screened out the high-risk groups of job burnout. It is necessary to pay more attention nurses who are single and working in general hospitals with many night shifts, especially nurses with severe PTSD. Hospitals can set up nurses’ personal health records to give timely warnings to nurses with health problems, and carry out support interventions to relieve occupational stress.

**Supplementary Information:**

The online version contains supplementary material available at 10.1186/s12912-024-01714-5.

## Introduction

In public health emergencies, nurses were often overworked in order to respond to medical emergencies [1]. Prolonged stress at work could lead to the burnout syndrome in nurses, which was defined as prolonged response to chronic emotional and interpersonal stressors at work and characterized by emotional exhaustion (EE), depersonalization (DP), and diminished personal accomplishment (PA) [2, 3]. The adverse factors of excessive workload and work risk push nurses into the population with a high incidence of job burnout [4].

Globally, job burnout is an important problem as it adversely affects the job performance [5]. Several systematic reviews and meta-analyses have previously demonstrated that nurses exhibit moderate to high levels of exhaustion syndrome. A 2023 systematic review including 29 articles consisting 16,619 healthcare workers showed 43% of healthcare workers suffered from burnout [6]. A meta-analysis covering over 30 countries in 2023 reported the global prevalence of nursing burnout was 30.0% [7]. A systematic review from Asia showed more than two third of healthcare providers suffered burnout during the COVID-19 pandemic [8]. As a populous country in Asia, China had a high prevalence of nursing burnout. A national survey of 51,406 registered nurses in China showed that half of them felt burnout [9]. A systematic review included 19 studies showed the pooled prevalence of burnout in mental health nurses were common. A survey with 1,061 nurses in Hunan province showed nurses experienced severe burnout symptoms [10]. Nurses maybe experience more severe burnout in departments of mental, oncology, and ICU [11–13]. In summary, nursing burnout is a frequent and serious health issue both globally and in China.

Nurses’ burnout varies between countries and regions. A 2021 Systematic review found compared with the Americas and Europe, Asian region had the highest levels of fatigue symptoms [14]. A large-scale survey in Taiwan province found the nurses had a moderate degree of emotional exhaustion in April, 2021. The mean score for emotional exhaustion in Liaoning, Guizhou and Hunan province were 11.74, 26.58 and 30, the dimensions of depersonalization and personal accomplishment also presented varying degrees of prevalence [5, 15, 16]. Therefore, it is of great significance to carry out epidemiological investigation of clinical nurse burnout in different regions of China in order to probe its risk factors.

The negative effects of nurse burnout were not only reflected in their own physical and mental health (such as the decline in the functioning of multiple organ systems, depression, anxiety, etc.), but also in the hospital organization and patients [17]. Job burnout can negatively impact health, including physical exertion, depression, low quality of life, and even high risk of suicide [18]. Also, job burnout may affect nurses’ behavior and attitude toward clinical service and increase the possibility of medical errors. Extended working hours during the pandemic could lead to burnout and other adverse psychological problems [19]. The psychological outcomes of healthcare workers during the pandemic have been studied globally. Common problems included burnout syndrome, anxiety, depression, and post-traumatic stress disorder (PTSD) [20]. Studies in Asia showed the varied proportions of these problems in each country: anxiety 8.7–90.0% and post-traumatic stress 2.1–9.1% [21–23]. Hence, to objectively and accurately reflect the burnout status of nurses respond to public health emergency, it is still necessary to investigate nurses’ burnout in different regions.

Most existing studies usually describe the status quo of nurse’ burnout using total score or pooled prevalence but the prediction of nurses’ burnout risk is neglected. Presently, a variety of statistical models were used to analyze risk factors and their relationships, but validation of the models was lacking. Consequently, this study aimed to conduct a cross-sectional survey among nurses in hospitals of Xiangyang City in order to describe the prevalence of nurses’ burnout and its three dimensions, as well as develop a well-functioning nomogram to predict their risk.

## Materials and methods

### Study design and setting

A cross-sectional survey was conducted by the nursing department of Xiangyang Central Hospital in 10 hospitals relied on the Xiangyang Nursing Society in January, 2023.

### Sampling methods

This study used two-stage stratified cluster random sampling to select participants. Firstly, hospitals were divided into two layers based on specialty and general hospitals, and 5 hospitals were selected from each level. Secondly, two third of the departments were randomly selected in the selected 10 hospitals, and nurses in the departments meeting the criteria were included in the study.

The sample size was calculated as follows:

$$ N=deff\frac{{\mu }^{2}p(1-p)}{{d}^{2}}$$

The confidence interval level was two-sided 95%, namely, *μ* = 1.96, *deff* = 1.5, and the allowable error *d* = 0.15*p*. Referring to previous literature, the positive rate of nurses’ job burnout ranged from 24 to 93.5%. To increase the sample size appropriately and ensure the research results, the *P*-value of this study was 30%, so the sample size was calculated to be 598 cases. Allowing for 20% of invalid questionnaires, the sample size was at least 717 cases.

### Target population

The target population was nurses who worked in hospital in Xiangyang City.

The inclusion criteria were as follows: (a) nurse with a national qualification certificate for nurse practitioners; (b) Regular staff of the hospital; (c) informed consent and willingness to participate in the study. The exclusion criteria were as follows: (a) Absence from work due to leave or study during the investigation period; (b) Received psychological or medical treatment within three months; (c) Disagree to participate in this study.

The questionnaire was developed through an online survey tool-WJX (www.wjx.cn). After repeated self-testing and revision by members of the survey team, respondents completed the questionnaire by accessing the QR code of the questionnaire through the WeChat APP. No mention of data cleaning and normality testing.

### Instruments

The online survey tool-WJX (www.wjx.cn) was used to prepared the questionnaire, which was repeatedly tested and revised by the members of the survey team. Then, the respondents completed the questionnaire by accessing the QR code of the questionnaire through the WeChat APP. All the raw data could be exported by WJX, sorted and cleaned by EXCEL. A total of 1605 questionnaires were collected by the WJX, but there were missing data and error message. We performed data cleaning through the following process. For more missing and uncorrectable data, we directly deleted; For less missing data, we tried to contact the respondents to supplement. If the respondents could not be contacted, the mean interpolation method was performed using Excel. After data cleaning, a total of 1584 valid questionnaires were collected in this study.

The questionnaire for this study was composed of three parts: (a) general information; (b) the Impact of Event Scale-Revised (IES-R); (c) the Chinese version of Maslach Burnout Inventory-General Survey (MBI-GS). The general information included the demographic variables (age, sex, marital status, fertility status, education level, etc.) and work-related variables (the length of service, professional title, the type of hospital, the number of night shifts per month, patients with infectious diseases, and so on).

The IES-R measure is an easily self-administered questionnaire to assess the symptoms of Post-Traumatic Stress Disorder (PTSD) following traumatic events. The IES-R is a widely used self-report measure feasible to assess traumatic stress caused by public health emergencies. The IES-R has 22 items, and this 22-item scale is factorized into three dimensions, namely: intrusion with eight items, 1, 2, 3, 6, 9, 14, 16, and 20; avoidance with eight items, 5, 7, 8, 11, 12, 13, 17, and 22; hyperarousal with six items, 4, 10, 15, 18, 19, and 21. The IES-R is designed with five Item Response Anchors rated from 0 to 4, where 0 indicates not at all; 1 = a little bit; 2 = moderately; 3 = quite a bit; and 4 = extremely [24]. Subsequently, the 22-item IES-R scores range from 0 to 88. The score of PTSD was obtained by adding the score of avoidance scale and invasion scale. In this study, PTSD was divided into four level: Not at all (0–8 scores), a little bit (9–25 scores), moderately (26–43 scores), quite a bit (more than 44 scores).

The Maslach Burnout Inventory-General Survey (MBI-GS) contains 16 items with three dimensions: emotional exhaustion (EE, five items), depersonalization (DP, four items), and diminished personal accomplishment (PA, six items), is used widely to measure the burnout syndrome [2]. In 2002, Professor Chao-Ping Li of Renmin University of China was authorized from Professor Michael Leiter, the developer of the MBI-GS, and then revised the scale and introduced it to China. The process of introducing the Chinese version of MBI-GS was as follows: firstly, Professor Li invited four experts to independently translate the original scale into Chinese, and then invited employees from different industries to conduct interviews, and then formed the first draft after repeated revisions. Secondly, it was translated into English by two English-speaking experts and sent back to the developer, Professor Michael Leiter, for comparison and modification, and then adjusted according to his comments, finally forming the Chinese version of the 16-item scale. Finally, the pre-survey and exploratory validation analyses of burnout among employees from three companies revealed that one item of “depersonalization” had a high cross load. After deleting this item, the results showed that the adjusted MBI-GS was completely consistent with the original MBI-GS structure. After the above process of sinicisation, the Chinese version of the 15-items MBI-GS with good reliability and validity was finally formed in China [25]. The revised 15-items scale has been proved to have good reliability and validity by many studies and is more localized in China [26–28], so we used the Chinese version of MBI-GS revised by Professor Li in our study.

Referring to the most commonly used criteria for the classification of job burnout in previous studies, 27 points, 10 points, and 15 points were used as the demarcation points of EE, DP, and PA of job burnout. The score of EE was more than 27 points, and the score of DP was more than 10 points. If the score of PA is more than 15 points, it is considered that the individual shows job burnout in the corresponding dimension. The burnout for the logistic regression model was divided into positive and negative by considering 50 scores as the boundary.

The reliability analysis and the factor analysis were used to test the reliability and validity of the instruments, which could be completed by the software IBM SPSS 29.0. The reliability of IES-R and MBI-GS was evaluated by Cronbach’s alpha coefficient and McDonald’s Omega, and the validity was evaluated by KMO and Bartley spherical test. The Cronbach’s alpha, McDonald’s Omega and KMO value of IES-R were 0.964, 0.964, and 0.974, respectively. The values were 0.901, 0.845, and 0.928 for the scale MBI-GS. The coefficient values of different dimensions of two scales were all more than 0.850. So, the scale IES-R and MBI-GS had good reliability and validity (Table 1s in the supplement).

### Statistical analysis

The Chi-square test compared categorical variables expressed as numbers and proportions. To develop a well-calibrated nomogram to predict the pathological outcomes, we performed univariate and multivariate logistic regression analyses to screen for predictors. A univariate analysis was performed to explore burnout response-related variables. Subsequently, multivariate logistic regression analysis was used to determine the independent influencing factors of burnout outcomes and develop the nomogram of the prediction model. A calibration method and the area under the receiver operating characteristic (ROC) curve were used to validate the nomogram internally.

Statistical analyses were performed using R version 4.2.3 software. A *P* value < 0.05 was considered to indicate statistical significance.

## Results

### The levels of PTSD when nurses faced the public health emergencies

A total of 1584 nurses in the hospital responded to the survey. Most respondents across the positions mentioned above were female (98.1%). The Impact of Events Scale-Revised (IES-R) showed that only 3.7% of nurses were completely free of PTSD during a public health emergency. We found that PTSD varied by age, marital status, procreation status, service year, employee status, and ICU. The nurses aged 30 ~ 40 years had higher levels of PTSD compared to those under 30 and over 40 years. Single and married nurses were more likely to have post-traumatic stress. The nurses who were married without children, single, with non-regular employees, worked for less than three years, or worked in the ICU had higher levels of PTSD. Table 1 summarizes the characteristics of related factors.

**Table 1:** The distribution characteristics of nurses affected by public health emergencies [*n*(%)]

Variables	N (%)		The levels of PTSD				χ^2^	*P*
		0	1	2	3	4		
Sex								
Male	30(1.9)	4(13.3)	4(13.3)	8(26.7)	9(30.0)	5(16.7)	2.853^a^	0.092
Female	1554(98.1)	55(3.5)	164(10.6)	481(31.0)	640(41.2)	214(13.8)		
Age								
<30	700(44.2)	35(5.0)	84(12.0)	234(33.4)	249(35.6)	98(14.0)	15.775	< 0.001^*^
30 ~ 40	614(38.8)	20(3.3)	69(11.2)	179(29.2)	267(43.5)	79(12.9)		
≥ 40	270(17.0)	4(1.5)	15(5.6)	76(28.1)	133(49.3)	42(15.6)		
Marital status								
Others	23(1.5)	0(0)	4(17.4)	5(21.7)	12(52.2)	2(8.7)	8.676^a^	0.013^*^
Single	431(27.2)	24(5.6)	56(13.0)	143(33.2)	152(35.3)	56(13.0)		
Married	1130(71.3)	35(3.1)	108(9.6)	341(30.2)	485(42.9)	161(14.2)		
Procreation status								
No	544(34.3)	24(4.4)	74(13.6)	179(32.9)	198(36.4)	69(12.7)	9.159	0.002^*^
Yes	1040(65.7)	35(3.4)	94(9.0)	310(29.8)	451(43.4)	150(14.4)		
Education								
College or below	398(25.1)	12(3.0)	49(12.3)	138(34.7)	152(38.2)	47(11.8)	0.429	0.512
Bachelor or more	1186(74.9)	47(4.0)	119(10.0)	351(29.6)	497(41.9)	172(14.5)		
Service year								
<3	241(15.2)	16(6.6)	33(13.7)	85(35.3)	84(34.9)	23(9.5)	10.126	0.006^*^
3 ~ 10	637(40.2)	25(3.9)	68(10.7)	191(30.0)	261(41.0)	92(14.4)		
≥ 10	706(44.6)	18(2.5)	67(9.5)	213(30.2)	304(43.1)	104(14.7)		
Professional title								
Primary	1012(63.9)	41(4.1)	116(11.5)	326(32.2)	397(39.2)	132(13.0)	4.725	0.094
Intermediate	524(33.1)	16(3.1)	51(9.7)	153(29.2)	220(42.0)	84(16.0)		
Advanced	48(3.0)	2(4.2)	1(2.1)	10(20.8)	32(66.7)	3(6.3)		
Employee status								
Non-Regular	1324(83.6)	56(4.2)	153(11.6)	409(30.9)	523(39.5)	183(13.8)	13.903	< 0.001^*^
Regular	260(16.4)	3(1.2)	15(5.8)	80(30.8)	126(48.5)	36(13.8)		
Hospital								
Specialized	245(15.5)	4(1.6)	22(9.0)	97(39.6)	96(39.2)	26(10.6)	3.264	0.071
General	1339(84.5)	55(4.1)	146(10.9)	392(29.3)	553(41.3)	193(14.4)		
ICU								
No	1479(93.4)	55(3.7)	150(10.1)	455(30.8)	612(41.4)	207(14.0)	4.016	0.045^*^
Yes	105(6.6)	4(3.8)	18(17.1)	34(32.4)	37(35.2)	12(11.4)		
Patients with infectious disease								
No	149(9.4)	6(4.0)	21(14.1)	58(38.9)	50(33.6)	14(9.4)	1.924	0.165
Yes	1435(90.6)	53(3.7)	147(10.2)	431(30.0)	599(41.7)	205(14.3)		
Night shifts (per month)								
≤ 3	617(39.0)	29(4.7)	66(10.7)	205(33.2)	240(38.9)	77(12.5)	1.177	0.555
4 ~ 5	552(34.8)	19(3.4)	59(10.7)	178(32.2)	229(41.5)	67(12.1)		
≧ 6	415(26.2)	11(2.7)	43(10.4)	106(25.5)	180(43.4)	75(18.1)		
Total	1584(100.0)	59(3.7)	168(10.6)	489(30.9)	649(41.0)	219(13.8)		

Note: PTSD, Post-traumatic stress disorder; 0 indicates not at all, 1 = a little bit, 2 = moderately, 3 = quite a bit, and 4 = extremely. ^a^ Continuity correction

### The prevalence of nurses’ job burnout

Table 2 depicts the prevalence of three dimensions by MBI-GS, which contains 15 items. The cut-off points for the three dimensions of emotional exhaustion, depersonalization, and diminished personal accomplishment were 27 points, 10 points, and 15 points, respectively, based on the most frequently used criteria for the classification of job burnout in prior studies. If the score of the emotional exhaustion dimension was greater than 27 points, the score of the depersonalization dimension was greater than 10 points, and the score of the diminished personal accomplishment dimension was greater than 15 points, the individual was deemed to have job burnout in the corresponding dimension.

**Table 2:** The Prevalence of nurse’ job burnout in three dimensions [*n*(%)]

Variables	Dimension of burnout syndrome						≥ 2 types of high burnout	*P*-value
	High EE	*P*-value	High DP	*P*-value	High PA	*P*-value		
Total	392(27.4)		769(48.5)		295(18.6)		661(41.7)	
Sex								
Male	5(1.3)	0.300	13(1.7)	0.564	5(1.7)	0.781	12(1.8)	0.846
Female	387(98.7)		756(98.3)		290(98.3)		649(98.2)	
Age (Year)								
<30	174(44.4)	0.993	374(48.6)	< 0.001^*^	156(52.9)	0.003^*^	324(49.0)	0.002^*^
30 ~ 40	151(38.5)		291(37.8)		93(31.5)		244(36.9)	
≥ 40	67(17.1)		104(13.5)		46(15.6)		93(14.1)	
Marital status								
Others	2(0.5)	0.094	5(0.7)	< 0.001^*^	3(1.0)	0.046^*^	3(0.5)	< 0.001^*^
Single	117(29.8)		241(31.3)		97(32.9)		213(32.2)	
Married	273(69.6)		523(68.0)		195(66.1)		445(67.3)	
Procreation status								
No	150(38.3)	0.059	295(38.4)	0.001^*^	121(41.0)	0.007^*^	271(41.0)	< 0.001^*^
Yes	242(61.7)		474(61.6)		174(59.0)		390(59.0)	
Education								
College or below	82(20.9)	0.027^*^	180(23.4)	0.125	106(35.9)	< 0.001^*^	171(25.9)	0.564
Bachelor or more	310(79.1)		589(76.6)		189(64.1)		490(74.1)	
Service year								
<3	49(12.5)	0.113	110(14.3)	< 0.001^*^	56(19.0)	0.006^*^	103(15.6)	< 0.001^*^
3 ~ 10	172(43.9)		347(45.1)		131(44.4)		300(45.4)	
≥ 10	171(43.6)		312(40.6)		108(36.6)		258(39.0)	
Professional title								
Primary	241(61.5)	0.505	517(67.2)	0.008^*^	212(71.9)	0.005^*^	444(67.2)	0.056
Intermediate	139(35.5)		236(30.7)		78(26.4)		201(30.4)	
Advanced	12(3.1)		16(2.1)		5(1.7)		16(2.4)	
Employee status								
Non-Regular	326(83.2)	0.795	668(86.9)	< 0.001^*^	255(86.4)	0.142	573(86.7)	0.005^*^
Regular	66(16.8)		101(13.1)		40(13.6)		88(13.3)	
Hospital								
Specialized	29(7.4)	< 0.001^*^	103(13.4)	0.027^*^	61(20.7)	0.006^*^	82(12.4)	0.004^*^
General	363(92.6)		666(86.6)		234(79.3)		579(87.6)	
ICU								
No	373(95.2)	0.102	722(93.9)	0.422	284(96.3)	0.026^*^	626(94.7)	0.071
Yes	19(4.8)		47(6.1)		11(3.7)		35(5.3)	
Patients with infectious disease								
No	16(4.1)	< 0.001^*^	47(6.1)	< 0.001^*^	37(12.5)	0.041^*^	37(5.6)	< 0.001^*^
Yes	376(95.9)		722(93.9)		258(87.5)		624(94.4)	
Night shifts (per month)								
≤ 3	129(32.9)	< 0.001^*^	245(31.9)	< 0.001^*^	120(40.7)	0.571	211(31.9)	< 0.001^*^
4 ~ 5	127(32.4)		277(36.0)		95(32.2)		228(34.5)	
≧ 6	136(34.7)		247(32.1)		80(27.1)		222(33.6)	
PTSD								
Not at all	2(0.5)	< 0.001^*^	3(0.4)	< 0.001^*^	18(6.1)	0.005^*^	4(0.6)	< 0.001^*^
A little bit	7(1.8)		25(3.3)		41(13.9)		21(3.2)	
Moderately	52(13.3)		154(20.0)		96(32.5)		116(17.5)	
Quite a bit	188(48.0)		395(51.4)		98(33.2)		343(51.9)	
Extremely	143(36.5)		192(25.0)		42(14.2)		177(26.8)	

Note: PTSD, Post-traumatic stress disorder; EE: Emotional exhaustion, DP: Depersonalization, PA: Diminished personal accomplishment. *: *P* < 0.05, the significant difference

Regarding the prevalence of burnout, 27.4%, 48.5%, and 18.6% of nurses had a high level of EE, DP, and PA, respectively. More than two types of job burnout were present in 41.7% of nurses. These figures of nurses, female, young, married, with children, higher level of education, longer service years, primary title, regular employee, worked in a general hospital, with three levels of PTSD had higher burnout syndrome. Marital status, procreation status, education, the types of hospitals, whether the department treats patients with infectious diseases, the number of night shifts (per month), and the level of PTSD were associated with EE. Age, marital status, procreation status, service years, employee status, the type of hospitals, the type of department, the number of night shifts (per month), and the level of PTSD were associated with DP. Age, marital status, procreation status, level of education, service year, professional title, types of hospital, type of department, and level of PTSD were associated with PA.

### The results of nurses’ job burnout and its three dimensions by logistic regression model

Tables 3 and 4 depict the results of univariate and multivariate logistic regression analysis. The burnout was divided into positive and negative by taking 50 scores as the boundary. This study found that the prevalence of nurse job burnout was 26.7%. A univariate analysis found that all factors except sex were associated with burnout.

**Table 3:** Univariate analysis of influencing factors of nurses’ job burnout

Variables	Total	Burnout		χ^2^	*P*
		No	Yes		
Total	1584(100.0)	1146(72.3)	438(27.7)	—	—
Sex					
Male	30(1.9)	25(2.2)	5(1.1)	1.844	0.174
Female	1554(98.1)	1121(97.8)	433(98.9)		
Age					
< 30	700(44.2)	467(40.8)	233(53.2)	21.448	< 0.001^*^
30 ~ 40	614(38.8)	464(40.5)	150(34.2)		
≥ 40	270(17.0)	215(18.8)	55(12.6)		
Marital status					
Others	23(1.5)	22(1.9)	1(0.2)	25.231	< 0.001*
Single	431(27.2)	276(24.1)	155(35.4)		
Married	1130(71.3)	848(74.0)	282(64.4)		
Procreation status					
No	544(34.3)	352(30.7)	192(43.8)	24.191	< 0.001*
Yes	1040(65.7)	794(69.3)	246(56.2)		
Education					
College or below	398(25.1)	293(25.6)	105(24.0)	0.428	< 0.001*
Bachelor or more	1186(74.9)	853(74.4)	333(76.0)		
Service year					
< 3	241(15.2)	170(14.8)	71(16.2)	23.086	< 0.001^*^
3 ~ 10	637(40.2)	424(37.0)	213(48.6)		
≥ 10	706(44.6)	552(48.2)	154(35.2)		
Professional title					
Primary	1012(63.9)	706(61.6)	306(69.9)	10.442	0.005^*^
Intermediate	524(33.1)	400(34.9)	124(28.3)		
Advanced	48(3.0)	40(3.5)	8(1.8)		
Employee status					
Non-Regular	1324(83.6)	937(81.8)	387(88.4)	10.041	0.002^*^
Regular	260(16.4)	209(18.2)	51(11.6)		
Hospital					
Specialized	245(15.5)	202(17.6)	43(9.8)	14.780	< 0.001*
General	1339(84.5)	944(82.4)	395(90.2)		
ICU					
No	1479(93.4)	1066(93.0)	413(94.3)	0.830	< 0.001^*^
Yes	105(6.6)	80(7.0)	25(5.7)		
Patients with infectious disease					
No	149(9.4)	125(10.9)	24(5.5)	10.956	< 0.001^*^
Yes	1435(90.6)	1021(89.1)	414(94.5)		
Night shifts (days per month)					
≤ 3	617(39.0)	488(42.6)	129(29.5)	35.528	< 0.001^*^
4 ~ 5	552(34.8)	400(34.9)	152(34.7)		
≧ 6	415(26.2)	258(22.5)	157(35.8)		
PTSD					
Not at all	3.7(3.7)	58(5.1)	1(0.2)	266.416	< 0.001^*^
A little bit	10.6(10.6)	156(13.6)	12(2.7)		
Moderately	30.9(30.9)	423(36.9)	66(15.1)		
Quite a bit	41(41.0)	432(37.7)	217(49.5)		
Extremely	13.8(13.8)	77(6.7)	142(32.4)		

Note: PTSD, Post-traumatic stress disorder; *: *P* < 0.05, the significant difference

**Table 4:** Univariate analysis of influencing factors of nurses’ job burnout

Variables	β	χ^2^	*P*-value	OR (95%CI)
Intercept	-8.005	—	—	—
Sex				
Male	1			
Female	1.040	3.544	0.060	2.829 (1.023 ~ 9.261)
Marital status				
Others				
Single	2.539	5.754	0.016^*^	12.665 (2.39 ~ 234.911)
Married	1.871	3.210	0.073	6.496 (1.278 ~ 118.873)
Service year				
<3	1			
3 ~ 10	0.284	1.756	0.185	1.328 (0.875 ~ 2.025)
≥ 10	-0.178	0.508	0.476	0.837 (0.515 ~ 1.368)
Hospital				
Specialized	1			
General	0.597	9.414	0.002^*^	1.816 (1.251 ~ 2.685)
Night shifts (days per month)				
≤ 3	1			
4 ~ 5	0.062	0.142	0.707	1.063 (0.772 ~ 1.466)
≧ 6	0.439	6.823	0.009^*^	1.550 (1.116 ~ 2.155)
PTSD				
Not at all	1			
A little bit	2.124	1.538	0.145	4.654 (0.879 ~ 85.989)
Moderately	2.328	5.209	0.022^*^	10.255 (2.178 ~ 183.302)
Quite a bit	12.263	3.555	< 0.001^*^	35.005 (7.549 ~ 622.993)
Extremely	4.891	22.863	< 0.001^*^	133.107 (28.016 ~ 2386.327)

Note: PTSD, Post-traumatic stress disorder; *: *P* < 0.05, the significant difference

Multivariate analysis revealed that the status of marriage, the type of hospital, the number of night shifts, and the severity of PTSD were included as job burnout risk factors for nurses. The single nurses had a higher risk of burnout (OR _single_=12.665). The nurses who worked in the general hospital and worked more than six days per month had a higher risk (OR _general_=1.816 and OR_≥ 6 days=1.550_). The risk of burnout increased with the level of PTSD (OR _Moderately_ =10.255, OR _Quite a bit_ =35.005, and OR _Extremely_ =133.107) (Table 4).

Similarly, logistic regression was performed for the three dimensions of job burnout (Table 2s in the supplement). We found that marriage status, the type of hospital, the night shifts, infectious patients, and PTSD were related with EE. The sex, marriage status, night shifts, infectious patients, service years, and PTSD were related with DP. Only the level of education, infectious patients, and working in the ICU were related with the PA.

**Fig. 1:**
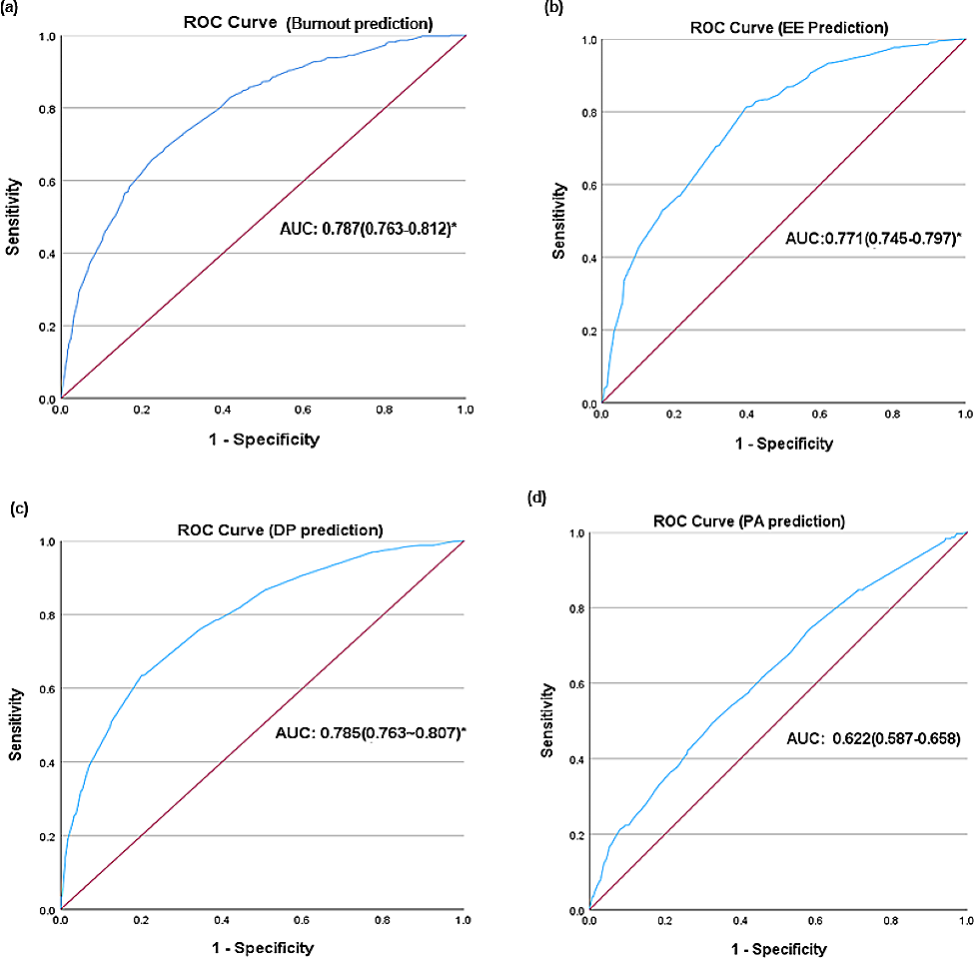
Receiver operating characteristic curve performance of logistic regression model for nurses’ job burnout and its three dimensions. (EE: Emotional exhaustion; DP: Depersonalization; PA: Diminished personal accomplishment; AUC: area under the curve.)

We used a receiver operating characteristic (ROC) curve, specificity, sensitivity, accuracy, and specificity/sensitivity to evaluate the prediction model in both validation and test data. Before the evaluation, optimal cutoffs were determined by maximizing the Youden index (i.e., sensitivity + specificity-1) using the ROC curve in the validation set. In this study, ROC curves revealed that logistic regression had better predictive performances. The area under curve for burnout and its three dimensions were 0.787, 0.771, 0.785, and 0.622, respectively, of which the predictive effect of PA was marginal (Fig. 1).

### The development of the nomogram and the validation of the calibration curve

**Fig. 2:**
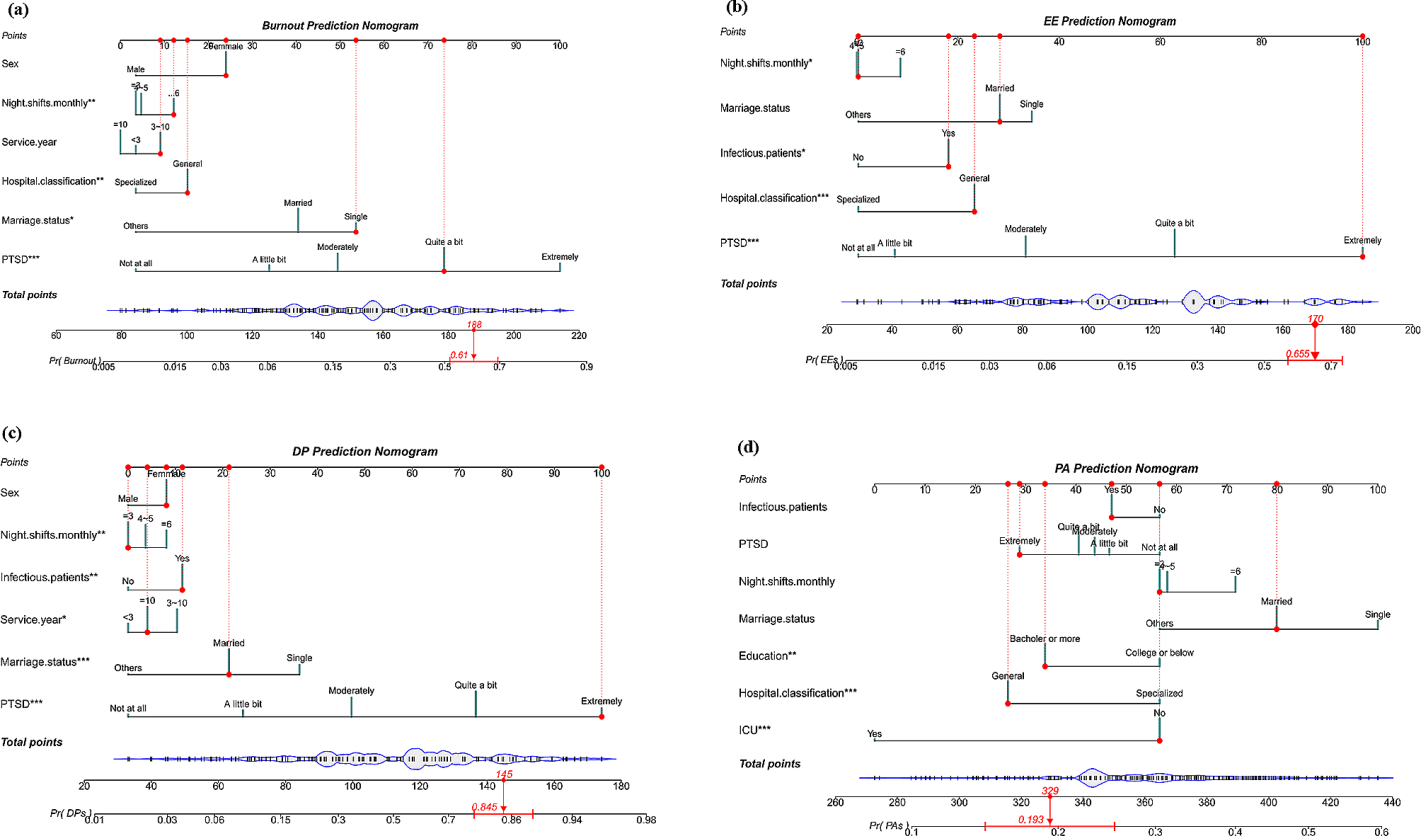
Developed nomogram with risk factors of nurses’ job burnout and its three dimensions. (EE: Emotional exhaustion, DP: Depersonalization, PA: Diminished personal accomplishment.)

As the prediction developed by logistic regression performed well, four nomograms (Fig. 2.) were used to present the data vividly and predicted probabilities. In Fig. 2a, a spike has been used to represent the proportion of the sample in category variables. In terms of gender, the majority of participants were classified as “female.” The total score corresponded to the bean-shaped predicted probability. For example, an individual score of 188 points in total, with a sex score of 24, six days night shifts per month score of 12, a length of service 3 ~ 10 years score of 9, worked at a general hospital score of 15, a single married status score of 54, a level of PTSD (quite a bit) score of 74 would have a 61.0% predicted probability of burnout. The same is true for other nomograms.

**Fig. 3:**
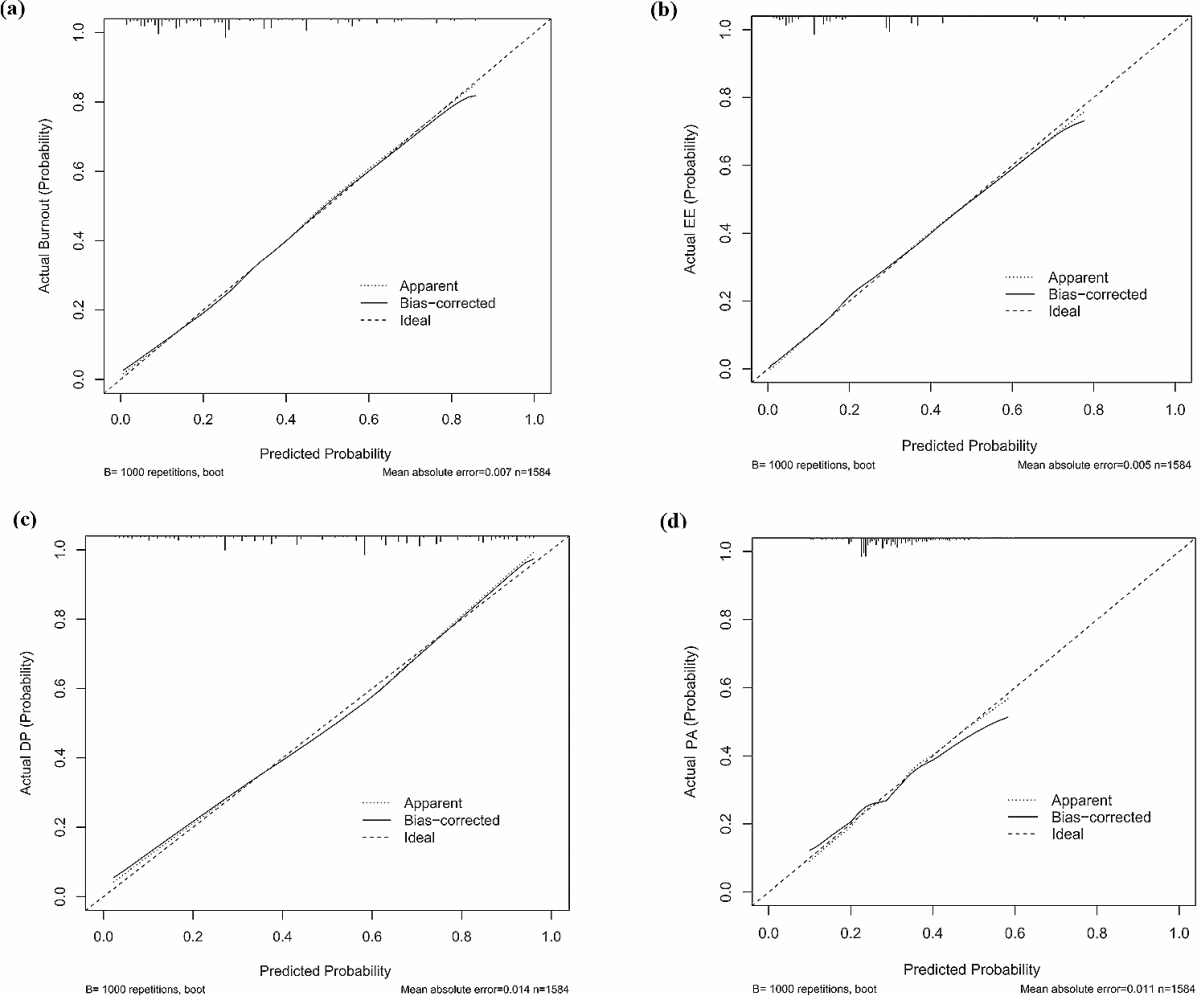
Calibration of the nomogram to predict nurses’ job burnout outcomes and its three dimensions. (EE: Emotional exhaustion, DP: Depersonalization, PA: Diminished personal accomplishment.)

The nomogram was calibrated (Fig. 3.) using a calibration plot with bootstrap sampling (*n* = 1000). According to the Hosmer-Lemeshow test, the predicted probabilities of EE, DP, and burnout were consistent with the actual probabilities, except for PA.

## Discussion

In this study, we found most nurses suffered from different degrees of PTSD during public health emergencies. The nursing burnout were associated with the number of night shifts, the type of hospital, marital status, and the degrees of PTSD.

This study found that only 3.7% of nurses were completely free of PTSD during public health emergencies, which showed nurses are vulnerable people in public health emergencies. We also found PTSD was highly correlated with job burnout, consistent with other studies [29, 30]. A study in South Korea found that because of the increased workloads during the COVID-19 pandemic, healthcare workers were leaved in exhaustion, which even in turn threatened their mental health [31]. In order to reduce the fatigue and trauma of nurses during public health emergencies, the inclusion of telenursing care services in the nursing education curriculum could make the existing health resources more rational use and improve health efficiency [32].

This study found that the nurses’ job burnout was associated with all three dimensions, explaining a 27.4% variance in emotional exhaustion, 48.5% in depersonalization, and 18.6% in personal accomplishment. Compared with other cross-sectional surveys (16% for EE, 13% for DP, and 10% for PA), the nurses in this study suffered greater burnout [33]. However, the prevalence of nurse’ job burnout was lower than earlier at the beginning of the epidemic [34]. Of course, it cannot be ruled out that there are differences in the medical system and the working environment for nurses in foreign countries. The first and foremost reason may be that nurses take more risks and work stress during public health emergencies [35, 36]. This also causes an increased workload on nurses and later results in a decrease in the quality of nursing care, threatens the safety of patients, and increases workforce-related mental health issues [37].

The present study found that nurses in general hospitals were more likely to be exposed to job burnout. The primary reasons may be that compared with specialized hospitals, general hospitals have a large number of outpatients and emergency patients, more inpatients, and general hospitals undertake the task of receiving patients referred by specialized hospitals [38, 39]. Such patients are generally complicated to deal with, nursing is difficult, and therefore, the nurses are under immense pressure. Furthermore, the requirements of nursing research, development of new technology, and business in general hospitals are much higher than those in specialized hospitals. Furthermore, nurses in general hospitals perform more examination and assessment activities than their colleagues in subordinate hospitals, which indirectly adds to job burnout. The job burnout of unmarried nurses was significantly higher than that of married staff. The possible reason is that the nursing work is complicated and heavy [40]. Married nurses were suffered from more burnout than the unmarried, which was consistent with other study [41]. The reason maybe the unmarried nurses are mostly young, have no clear plan for career development goals and careers, lack work experience, face frequent assessment and research pressure, and emotional instability. The cause of job burnout may be related to the gap between job expectations and professional reality, and the lack of work experience leading to a low fit with the post is also one of the important reasons for aggravating job burnout.

Nurses who had worked for 3–10 years were prone to emotional exhaustion. Nurses with 3–10 years of nursing experience were mostly the department’s backbone and were in the rising stage of their own lives. They had to undertake a lot of nursing work in the department, complete the role of nurses, and also take the role of wife, mother, and daughter in the family, which was easy to form role conflict. The double pressure of career and family often makes nurses powerless, leading to increased job burnout. The service objects of nursing work are emotionally susceptible people, who often produce emotional excitement under the torture of the disease and the stimulation of the external environment. Nurses with 3–10 years of nursing experience were less enthusiastic with patients than young nurses with less than three years of nursing age and less steady [42]; thus, it was easy to be in a negative emotional state of irritability and irritability in frequent contact with service objects. Most nurses with 3–10 years of experience had well-defined career development objectives and career plans, aiming to maximize their professional skills and potential and realize their value. However, the distance between reality and expectation could easily lead to a “career plateau”, forming negative effects such as depression, loss of interest in work, and psychological burnout.

Depersonalization is common among nurses with more night shifts per month. This was because the special nature of work in the hospital requires nurses to implement the 24-hour shift system. This continuous and dynamic work made the nursing staff unable to go to work. According to normal circumstances, life was irregular, with shift work, night shift, and overtime work, and nurses were unable to eat and sleep on time, resulting in a disorder of their biological cycle and an increased likelihood of developing physical and mental exhaustion [43, 44]. The nursing staff was overburdened by insufficient family care and the interaction of family and work pressure [45].

Nurses working in the ICU had lower personal accomplishments. It may be that ICU work was risky and more susceptible to chronic fatigue that was affected by the intensity of clinical practice, the risk of litigation, and the disruption of circadian rhythms. Several studies have shown that the work department is the influential factor in job burnout, and the degree of job burnout of nurses in the ICU is more serious than that in general departments [46, 47]. Relatively more critical and severe cases occur in the ICU, where the patient’s condition is imperative, the progression is rapid, and the occupational risk is high. It needs nurses to have rapid response ability and high comprehensive quality.

The variability and uncertainty of public health emergencies lead to an increase in the workload of nurses beyond their ability to cope, and then leads to a range of serious physical and mental health problems [32]. How to reduce the nursing burnout in public health emergencies? The main recommendations are as follows: From an individual perspective, nurses themselves need to improve their competence (including professional competence, physical fitness, mental health, etc.) in order to cope with the variable demands of emergencies. For example, participating in physical exercise, adopting various ways to regulate and relieve their own stress, participating in professional training/continuing education, and enhancing emotional management [48–50]. From the hospital perspective, as managers, it is necessary to raise awareness of burnout and mental health. For example, the establishment of nurses’ personal health records and the provision of timely warnings to those with health problems [51]. According to the risk factors of job burnout, support interventions are carried out to relieve occupational stress. For example, interventions alleviating occupational stress such as rational allocation of health resources and improvement of working conditions.

### Limitations

There are some shortcomings in this study. First, this study used the self-report method of the subjects, which may have self-report bias. Second, this cross-sectional study cannot elaborate on the causal relationship between burnout and its related factors. Thirdly, due to the limitation of conditions, this study did not investigate all hospitals in Xiangyang City, and the results have certain limitations. The investigation scope can be expanded to obtain more solid data results. Fourth, this study focused only on the prevalence and the short-term effect of burnout, and did not use objective indicators of work and the long effects. In addition, intervention research can also be carried out to make practical exploration of the mental health of nurses. We recommend further expanding the sample size, optimizing the prediction model, and analyzing the interaction paths between the factors. Qualitative research of nursing burnout maybe carried out to further verify the conclusion. Furthermore, research on strengthening evidence-based evidence and supportive interventions to provide reference for caregivers’ occupational health.

## Conclusion

This study confirmed the PTSD and burnout are common problems for in-service nurses during public health emergencies and screened out the high-risk groups of job burnout. It is necessary to pay more attention nurses who are single and working in general hospitals with many night shifts, especially nurses with severe PTSD. Hospitals can set up nurses’ personal health records to give timely warnings to nurses with health problems, provide psychological intervention training to help nurses adjust the psychological state, and carry out support interventions to relieve occupational stress.

### Electronic supplementary material

Below is the link to the electronic supplementary material.

Supplementary Material 1: Table 1s The reliability and validity for the IES-R and MBI-GS. Table 2s Multivariate analysis of influencing factors of the three dimensions of job burnout

## Data Availability

Data used to support the findings of this study are available from the corresponding author upon request.
