# Supplementary material for: Risk and prediction of job burnout in responding nurses to public health emergencies
Source: BMC Nurs. 2024 Jan 17;23:46. doi: 10.1186/s12912-024-01714-5 (PMC10792923; doi:10.1186/s12912-024-01714-5)
Supplement: Supplementary file 1 — Supplementary Material 1: Table 1s The reliability and validity for the IES-R and MBI-GS. Table 2s Multivariate analysis of influencing factors of the three dimensions of job burnout [file 12912_2024_1714_MOESM1_ESM.docx]

**Table 1s** The reliability and validity for the IES-R and MBI-GS

| Scale | | KMO | Cronbach's α | McDonald’s ω |
| --- | --- | --- | --- | --- |
| IES-R | Int | 0.925 | 0.891 | 0.894 |
|  | Avo | 0.933 | 0.922 | 0.922 |
|  | Hyp | 0.914 | 0.913 | 0.913 |
|  | Total | 0.974 | 0.964 | 0.964 |
| MBI-GS |  |  |  |  |
|  | EE | 0.903 | 0.967 | 0.967 |
|  | DP | 0.856 | 0.958 | 0.956 |
|  | PA | 0.916 | 0.957 | 0.958 |
|  | Total | 0.928 | 0.901 | 0.845 |

Note: IES-R, The Impact of Events Scale-Revised; MBI-GS, The Maslach Burnout Inventory measured burnout syndrome. General Survey; KMO, Kaiser-Meyer-Olkin test statistic.

**Table 2s** Multivariate analysis of influencing factors of the three dimensions of job burnout

|  | EE | | | |  | DP | | | |  | PA | | | |
| --- | --- | --- | --- | --- | --- | --- | --- | --- | --- | --- | --- | --- | --- | --- |
|  | *β* | Wald | OR (95%CI) | *P* |  | *β* | Wald | OR (95%CI) | P |  | *β* | Wald | OR (95%CI) | P |
| Intercept | -6.942 | 29.387 | 0.001(0~0.010) | <0.001^*^ |  | -5.488 | 31.357 | 0.004(0.001~0.026) | < 0.001^*^ |  | 0.255 | 0.106 | 1.290(0.262~5.717) | 0.7445 |
| Sex |  |  |  |  |  |  |  |  |  |  |  |  |  |  |
| Male |  |  |  |  |  |  |  |  |  |  |  |  |  |  |
| Female | 0.523 | 0.826 | 1.687(0.583~5.751) | 0.363 |  | 0.392 | 0.681 | 1.480(0.584~3.800) | 0.409 |  | 0.045 | 0.010 | 1.046(0.455~2.630) | 0.9191 |
| Age |  |  |  |  |  |  |  |  |  |  |  |  |  |  |
| ＜30 |  |  |  |  |  |  |  |  |  |  |  |  |  |  |
| 30~40 | 0.176 | 0.590 | 1.193(0.759~1.870) | 0.442 |  | -0.340 | 2.707 | 0.712(0.474~1.068) | 0.100^*^ |  | 0.034 | 0.032 | 1.035(0.710~1.506) | 0.8572 |
| ≥40 | 0.081 | 0.051 | 1.084(0.536~2.182) | 0.821 |  | -0.869 | 7.297 | 0.42(0.223~0.787) | 0.007^*^ |  | 0.008 | 0.001 | 1.008(0.541~1.870) | 0.9787 |
| Marital status |  |  |  |  |  |  |  |  |  |  |  |  |  |  |
| Others |  |  |  |  |  |  |  |  |  |  |  |  |  |  |
| Single | 1.354 | 2.729 | 3.873(0.931~26.857) | 0.099 |  | 1.625 | 7.757 | 5.080(1.671~16.918) | 0.005^*^ |  | 0.274 | 0.238 | 1.315(0.464~4.354) | 0.6259 |
| Married | 1.257 | 2.560 | 3.513(0.918~23.277) | 0.110 |  | 1.052 | 3.821 | 2.864(1.039~8.830) | 0.051^*^ |  | 0.331 | 0.405 | 1.393(0.537~4.323) | 0.5246 |
| Procreation status |  |  |  |  |  |  |  |  |  |  |  |  |  |  |
| No |  |  |  |  |  |  |  |  |  |  |  |  |  |  |
| Yes | -0.590 | 4.872 | 0.555(0.33~0.942) | 0.027^*^ |  | -0.171 | 0.491 | 0.843(0.521~1.359) | 0.484 |  | -0.261 | 1.347 | 0.770(0.497~1.204) | 0.2458 |
| Education |  |  |  |  |  |  |  |  |  |  |  |  |  |  |
| College or below |  |  |  |  |  |  |  |  |  |  |  |  |  |  |
| Bachelor or more | 0.123 | 0.511 | 1.131(0.809~1.590) | 0.475 |  | -0.056 | 0.142 | 0.946(0.706~1.264) | 0.706 |  | -0.413 | 9.070 | 0.661(0.506~0.866) | 0.0026^*^ |
| Service year |  |  |  |  |  |  |  |  |  |  |  |  |  |  |
| ＜3 |  |  |  |  |  |  |  |  |  |  |  |  |  |  |
| 3~10 | 0.373 | 2.403 | 1.451(0.909~2.336) | 0.121 |  | 0.601 | 7.605 | 1.824(1.192~2.804) | 0.006^*^ |  | -0.008 | 0.002 | 0.992(0.679~1.450) | 0.9654 |
| ≥10 | 0.241 | 0.530 | 1.273(0.666~2.444) | 0.467 |  | 0.617 | 4.265 | 1.854(1.034~3.338) | 0.039^*^ |  | -0.201 | 0.544 | 0.818(0.479~1.397) | 0.4606 |
| Professional title |  |  |  |  |  |  |  |  |  |  |  |  |  |  |
| Primary |  |  |  |  |  |  |  |  |  |  |  |  |  |  |
| Intermediate | 0.290 | 2.025 | 1.336(0.898~1.995) | 0.155 |  | 0.135 | 0.565 | 1.145(0.805~1.630) | 0.452 |  | -0.207 | 1.393 | 0.813(0.576~1.146) | 0.2379 |
| Advanced | 0.240 | 0.286 | 1.271(0.515~3.018) | 0.593 |  | 0.155 | 0.151 | 1.167(0.533~2.550) | 0.698 |  | -0.402 | 0.762 | 0.669(0.257~1.590) | 0.3828 |
| Employee status |  |  |  |  |  |  |  |  |  |  |  |  |  |  |
| Non-Regular |  |  |  |  |  |  |  |  |  |  |  |  |  |  |
| Regular | 0.115 | 0.204 | 1.122(0.677~1.844) | 0.652 |  | -0.068 | 0.090 | 0.934(0.599~1.461) | 0.765 |  | -0.240 | 1.031 | 0.787(0.492~1.244) | 0.3099 |
| Hospital |  |  |  |  |  |  |  |  |  |  |  |  |  |  |
| Specialized |  |  |  |  |  |  |  |  |  |  |  |  |  |  |
| General | 0.893 | 15.233 | 2.442(1.582~3.888) | <0.001^*^ |  | 0.043 | 0.067 | 1.044(0.752~1.448) | 0.796 |  | -0.516 | 0.970 | 0.597(0.440~0.812) | <0.001^*^ |
| ICU |  |  |  |  |  |  |  |  |  |  |  |  |  |  |
| No |  |  |  |  |  |  |  |  |  |  |  |  |  |  |
| Yes | -0.350 | 1.365 | 0.705(0.382~1.244) | 0.243 |  | -0.153 | 0.393 | 0.858(0.531~1.386) | 0.531 |  | -0.982 | 1.721 | 0.375(0.206~0.640) | <0.001^*^ |
| Patients with infectious disease |  |  |  |  |  |  |  |  |  |  |  |  |  |  |
| No |  |  |  |  |  |  |  |  |  |  |  |  |  |  |
| Yes | 0.720 | 5.430 | 2.054(1.149~3.880) | 0.020^*^ |  | 0.596 | 7.394 | 1.814(1.185~2.800) | 0.007^*^ |  | -0.119 | 0.359 | 0.888(0.604~1.318) | 0.5489 |
| Night shifts (per month) |  |  |  |  |  |  |  |  |  |  |  |  |  |  |
| ≤3 |  |  |  |  |  |  |  |  |  |  |  |  |  |  |
| 4~5 | 0.036 | 0.040 | 1.037(0.728~1.477) | 0.842 |  | 0.088 | 0.331 | 1.092(0.809~1.475) | 0.565 |  | -0.161 | 1.188 | 0.851(0.636~1.137) | 0.2758 |
| ≧6 | 0.386 | 4.360 | 1.471(1.024~2.115) | 0.037^*^ |  | 0.320 | 3.700 | 1.377(0.994~1.909) | 0.054 |  | 0.090 | 0.331 | 1.094(0.805~1.486) | 0.5649 |
| PTSD |  |  |  |  |  |  |  |  |  |  |  |  |  |  |
| Not at all | 0.237 | 0.083 | 1.267(0.293~8.726) | 0.773 |  | 1.252 | 3.873 | 3.497(1.153~15.210) | 0.049^*^ |  | -0.175 | 0.276 | 0.839(0.440~1.632) | 0.5993 |
| A little bit | 1.320 | 3.196 | 3.743(1.106~23.393) | 0.074 |  | 2.459 | 16.451 | 11.694(4.172~48.867) | < 0.001^*^ |  | -0.190 | 0.393 | 0.827(0.461~1.521) | 0.5306 |
| Moderately | 2.508 | 11.837 | 12.286(3.727~75.937) | <0.001^*^ |  | 3.867 | 40.688 | 47.805(17.065~199.783) | < 0.001^*^ |  | -0.221 | 0.544 | 0.801(0.450~1.467) | 0.4609 |
| Quite a bit | 4.065 | 30.272 | 58.242(17.214~364.299) | <0.001^*^ |  | 5.227 | 67.048 | 186.287(61.573~814.854) | < 0.001^*^ |  | -0.420 | 1.638 | 0.657(0.348~1.265) | 0.2006 |

Note: PTSD, Post-traumatic stress disorder; *: *P* <0.05, the significant difference.
